# The type 2 high intensity zone: Two cases of axial low back pain in the setting of high T1 and high T2 signal in the posterior annulus

**DOI:** 10.1016/j.inpm.2022.100131

**Published:** 2022-09-06

**Authors:** Cyrus Ghaffari, Jeremy Heit, Josh Levin

**Affiliations:** a Department of Orthopaedic Surgery, Stanford University, 450 Broadway St, Pavilion C; 4th Floor, MC 6342, Redwood City, CA, 94063, USA; b Department of Radiology, Stanford University, 213 Quarry Rd, 4th Floor MC 5958, Palo Alto, CA, 94304, USA; c Department of Neurosurgery, Stanford University, 213 Quarry Rd, 4th Floor MC 5958, Palo Alto, CA, 94304, USA

Dear Editor,

The high intensity zone (HIZ) was first described by Aprill and Bogduk in 1992 [1]. The authors found that in patients with low back pain, a focal high intensity signal was often located in the posterior annulus fibrosis in one or more discs on T2-weighted MR images of the lumbar spine. Bogduk subsequently noted that the presence of an HIZ in a patient with low back pain was strongly suggestive of a discogenic etiology of the patient's pain, with an indicative likelihood ratio of 3.8. In other words, an investigator can be 73% certain that a patient presenting with low back pain who has an HIZ will have a painful response to a diagnostic provocation discography [2]. Pathoanatomic studies have shown that the majority of disc injuries occur due to avulsion at the junction of the endplate and the vertebral body [3]. This can begin a cascade of degenerative changes of the discovertebral complex that often presents as Modic changes on MRI [4].

MRI utilizes a magnetic field that causes hydrogen protons in water molecules to align. Next, the alignment is disrupted by an external radiofrequency (RF) source. The nuclei return to their resting alignment at different times and emit RF energy as they do so. Repetition time (TR - the amount of time between each pulse sequence), and time to echo (TE - the time between the delivery of an RF pulse and the collection of the echo signal) vary based on two different relaxation times, T1 and T2. This variation leads to differences in tissue characteristics. Fluid shows up bright on T2 and dark on T1, while fat shows up bright on both [5].

We present 2 cases of patients with bright signal in the posterior disc, not only on T2 weighted images, but also on T1 weighted images. In order to describe this phenomenon in an analogous manner to Modic changes, in which type 2 Modic changes demonstrate bright signal both on T1 and T2 weighted images [4], we refer to our finding as the type 2 HIZ.

The first patient was a 35-year-old woman with chronic episodic midline axial low lumbar pain, a history previously shown to be highly indicative of lumbar discogenic pain [6]. During an episode, any position could exacerbate her pain. At the time of her evaluation, her examination was unremarkable. MRI of the lumbar spine showed high T1 and T2 signal in the posterior L5-S1 disc (in the setting of a mostly lumbarized S1 segment) (Fig. 1, Fig. 2.) Fig. 3 shows a T2 fat suppression sagittal image at the same cut as the images seen in Fig. 1, Fig. 2, and there is only subtly increased signal at the same location. Fig. 4, however, shows a fat suppression image at the adjacent cut (just to the right of the image seen in Fig. 1, Fig. 2, Fig. 3) with more obvious increased signal. An axial image is not available to review as the axial cuts occurred just superior and inferior to this finding.

**Fig. 1:**
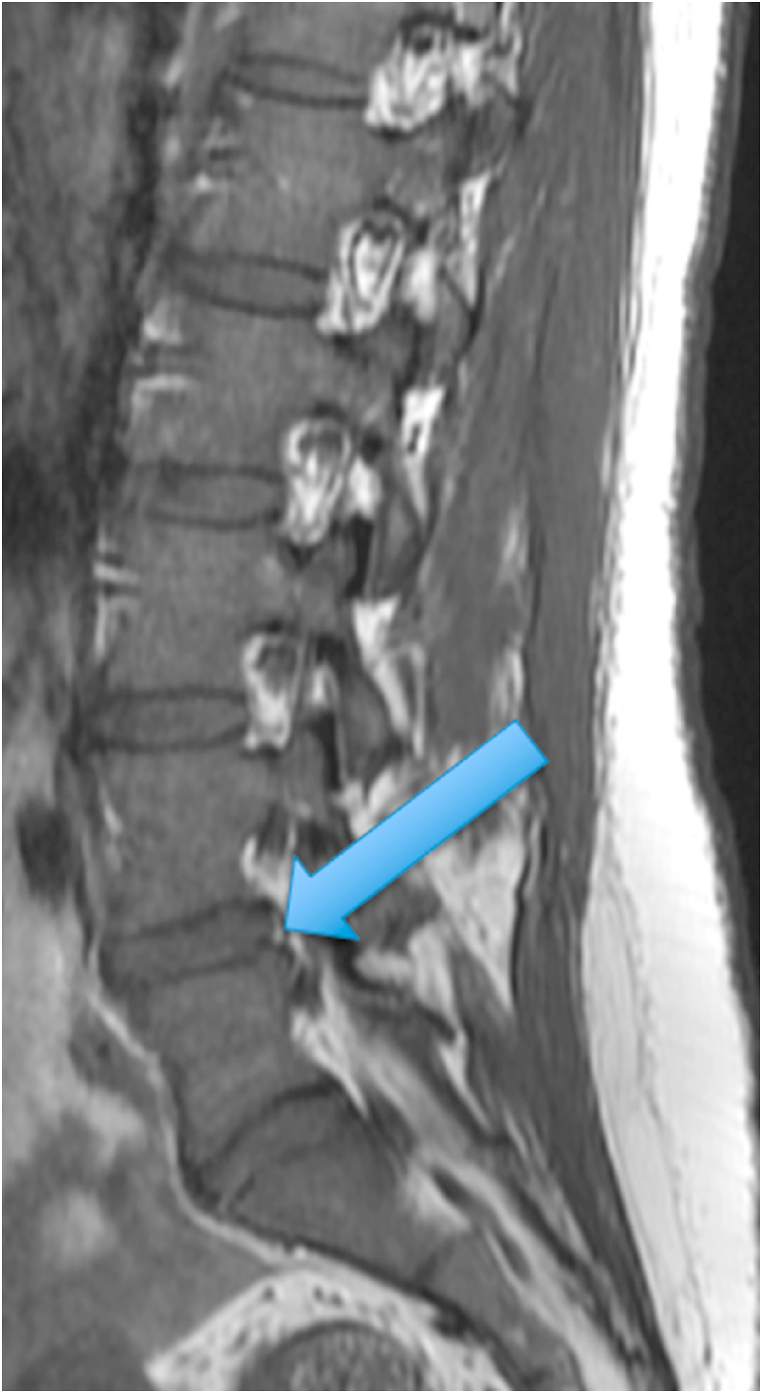
T1 sagittal image showing increased signal in the posterior L5-S1 disc (in the setting of a mostly lumbarized S1 segment).

**Fig. 2:**
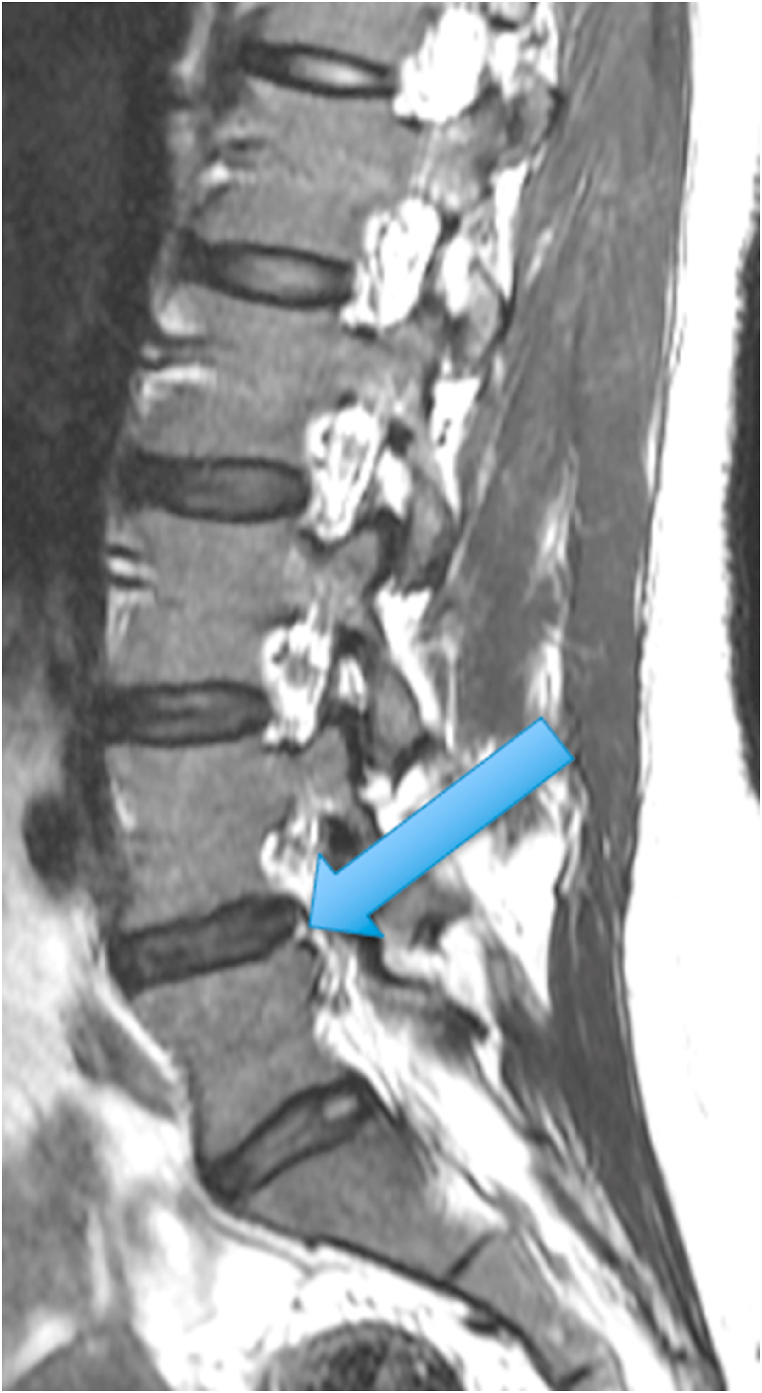
T2 sagittal image showing increased signal in the posterior L5-S1 disc (in the setting of a mostly lumbarized S1 segment).

**Fig. 3:**
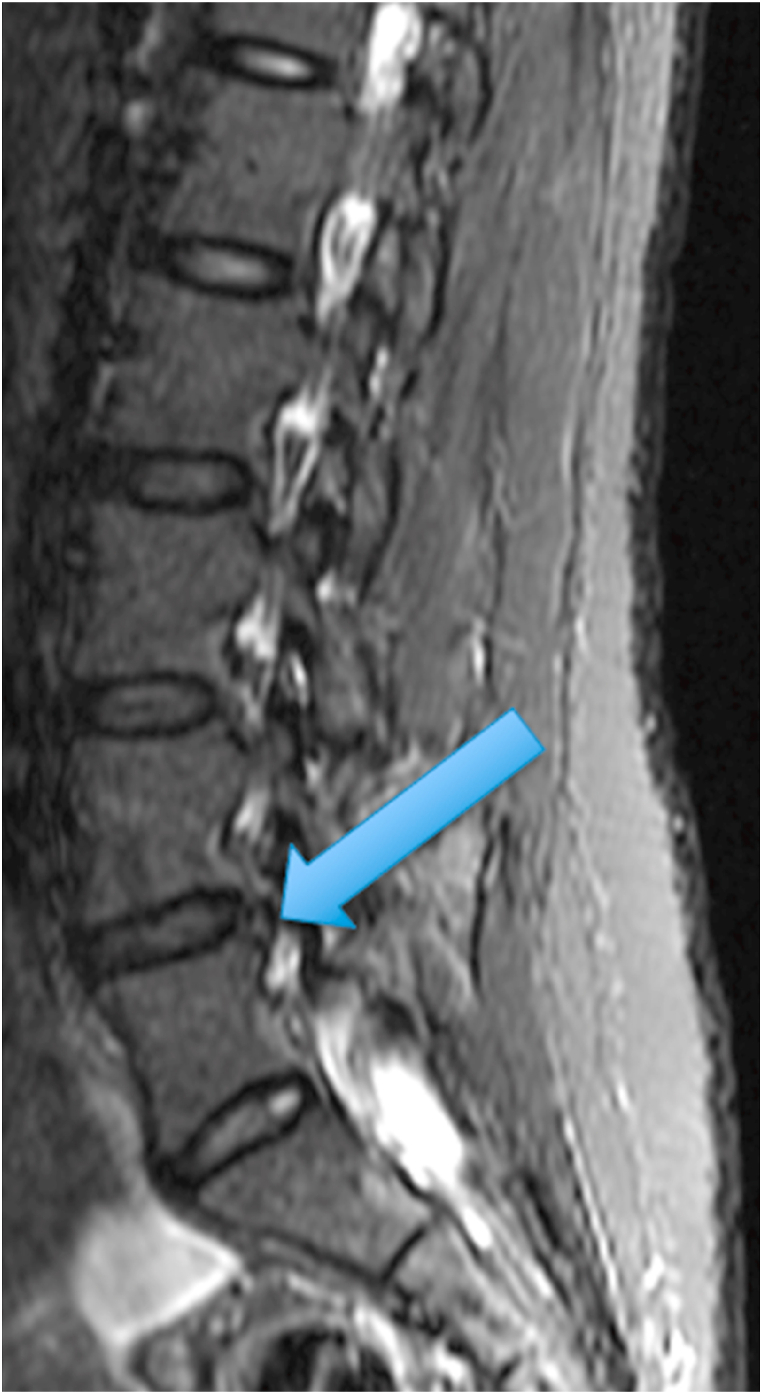
T2 fat suppression sagittal image at the same cut as the images seen in Fig. 1, Fig. 2 with subtly increased signal in the posterior L5-S1 disc (in the setting of a mostly lumbarized S1 segment).

**Fig. 4:**
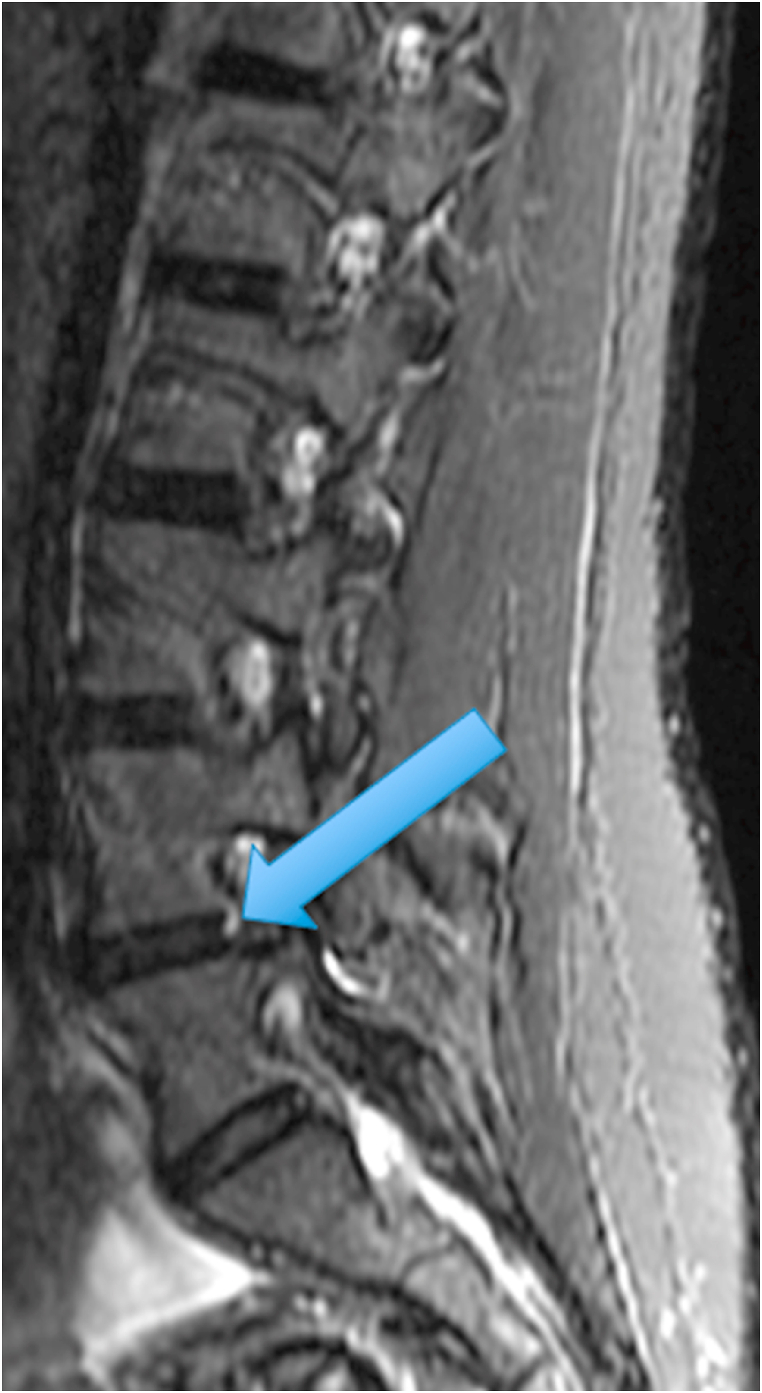
T2 fat suppression sagittal image at the adjacent cut just to the right of the images seen in Fig. 1, Fig. 2 showing increased signal in the posterior L5-S1 disc (in the setting of a mostly lumbarized S1 segment).

The second patient was a 71-year-old woman with a several year history of right sided low back pain referring into the right leg in an ill-defined location. She reported constant pain without specific exacerbating factors. Her examination was generally unremarkable. MRI of the lumbar spine showed high T1 and T2 signal in the posterior L3-L4 disc, in addition to type 2 Modic changes at this level (Fig. 5, Fig. 6.) Fig. 7 shows a T2 fat suppression sagittal image without increased signal in the posterior L3-4 disc. Fig. 8 shows a T2 axial image with subtly increased signal in the disc.

**Fig. 5:**
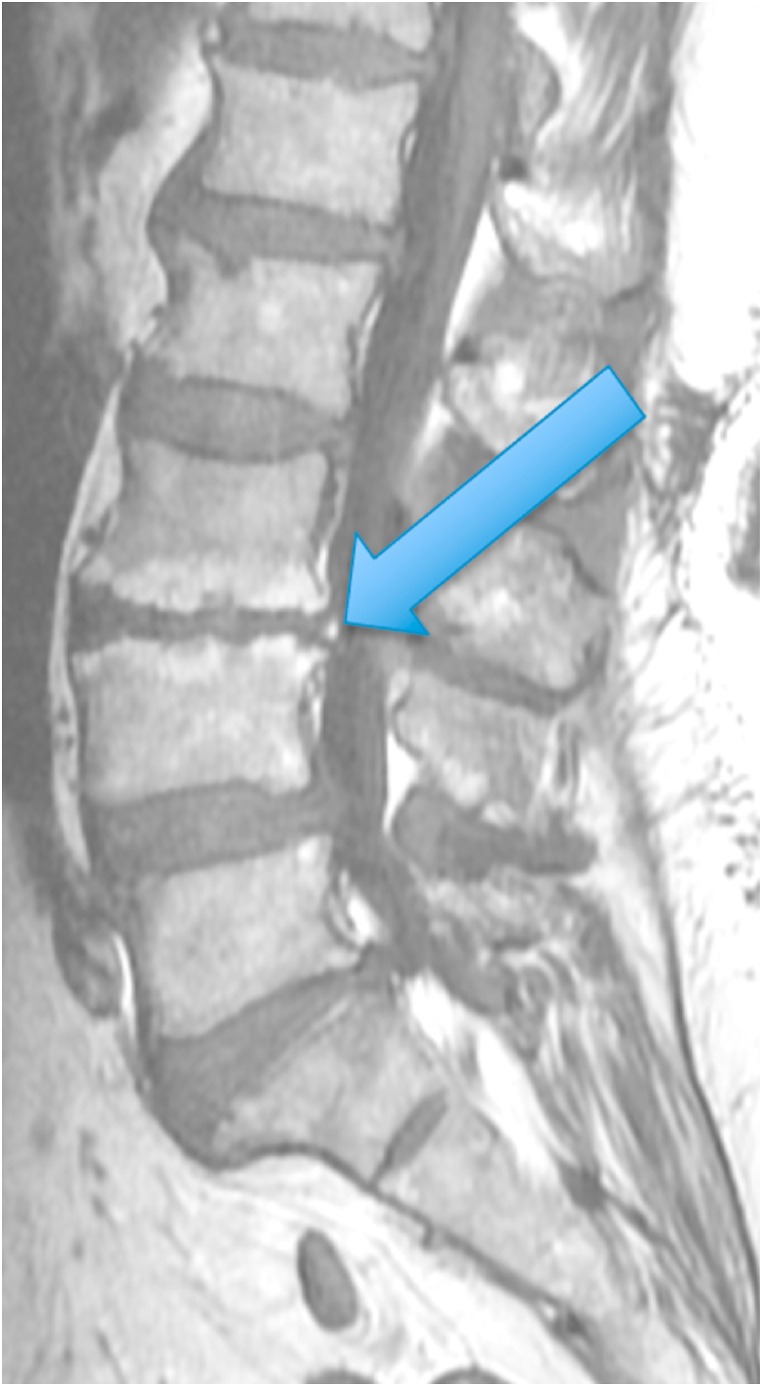
T1 sagittal image showing increased signal in the posterior L3-4 disc.

**Fig. 6:**
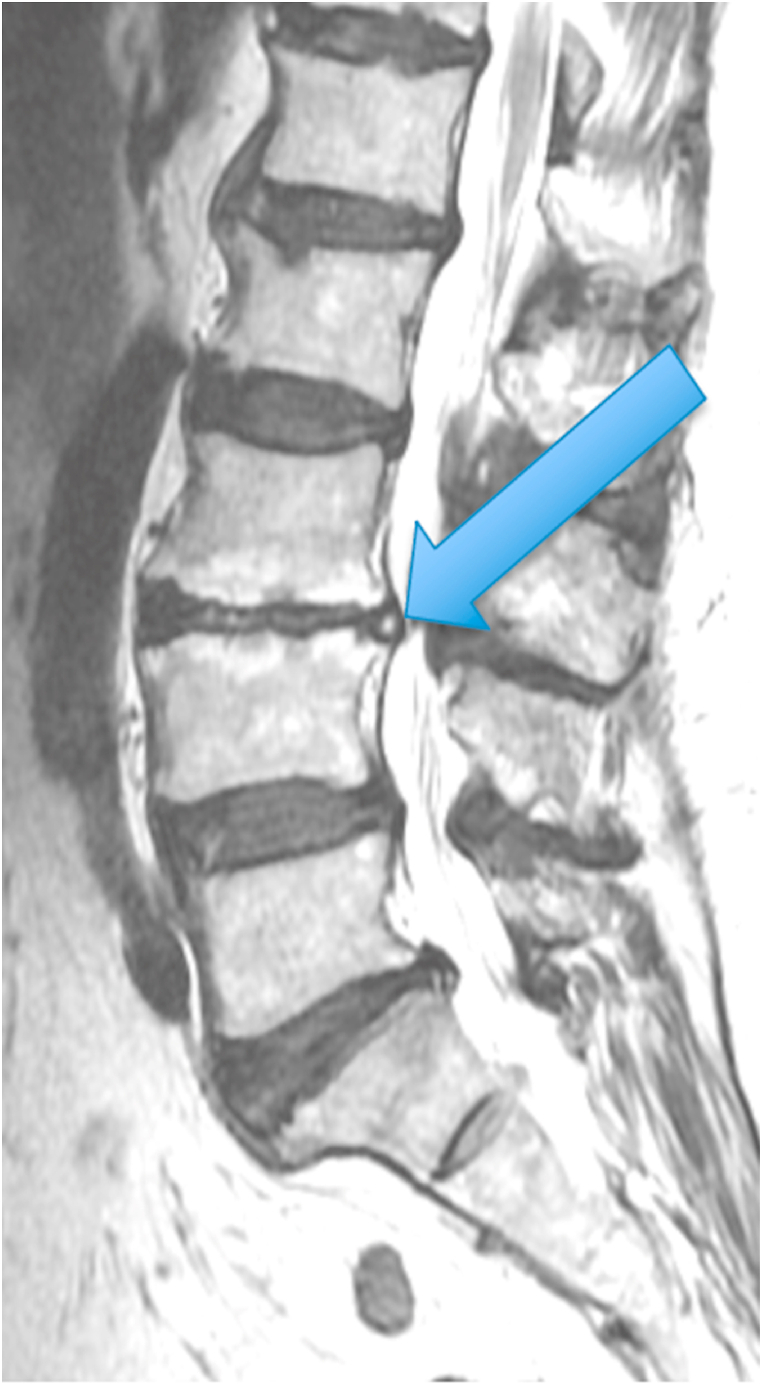
T2 sagittal image showing increased signal in the posterior L3-4 disc.

**Fig. 7:**
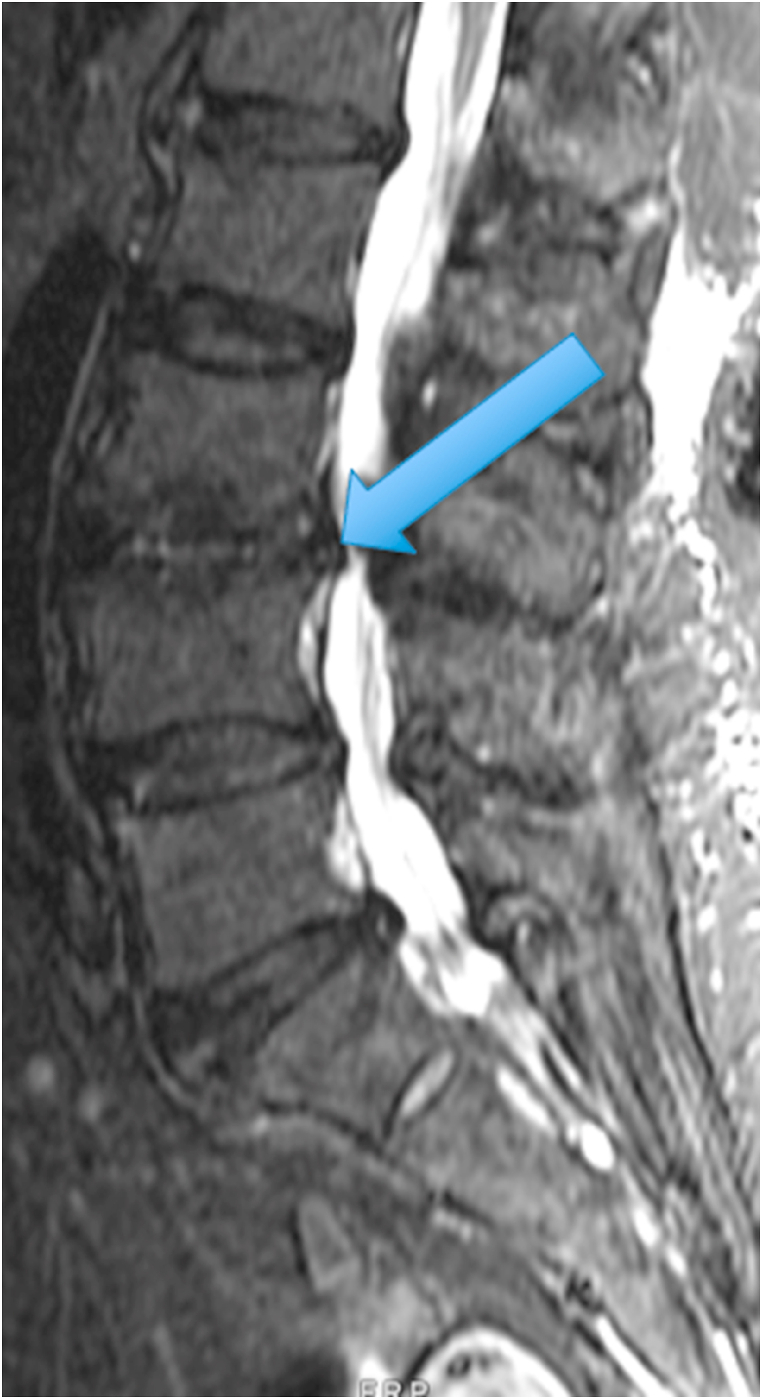
T2 fat suppression sagittal image without increased signal in the posterior L3-4 disc.

**Fig. 8:**
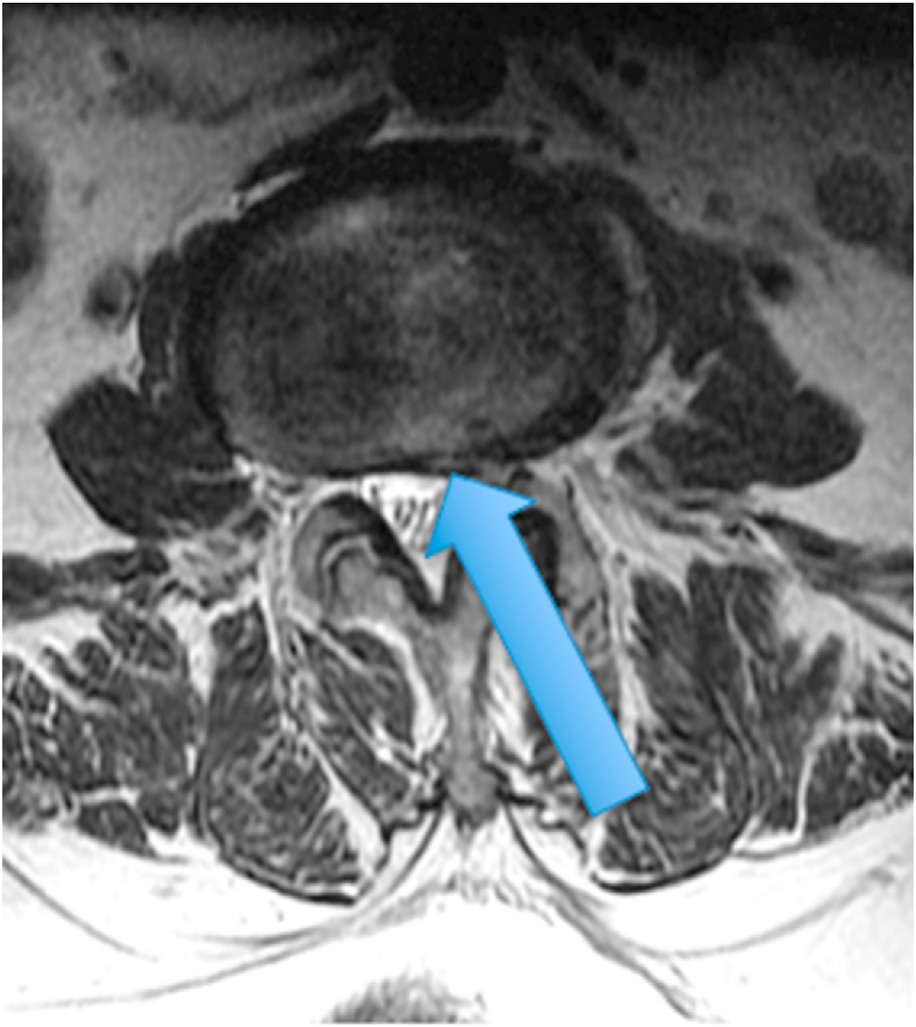
T2 axial image with subtly increased signal in the posterior L3-4 disc.

The two cases presented above demonstrate an MRI finding that we refer to as the type 2 high intensity zone. This finding may represent fatty change within the posterior disc, like fatty marrow changes seen in patients with type 2 Modic endplate changes. Alternatively, this imaging finding may represent hemorrhage in the disc, as this also can be bright on T1 and T2 in the late subacute stage of hemorrhage when extracellular methemoglobin is present [7]. The absence of clearly increased signal on the T2 fat suppression images in Fig. 3, Fig. 7 suggests that this could in fact be fat, however the increased signal on the T2 fat suppression image of the adjacent cut in the first patient (Fig. 4) suggests that hemorrhage may be more likely. Histologic studies would be required to provide a clear answer to this question, and additional clinical studies are required to determine the clinical significance of this finding.

Dr. Josh Levin reports a relationship with Scilex Pharmaceuticals that includes: consulting or advisory. The other authors report no relevant disclosures or conflicts of interest. The authors report no funding sources.

## Declaration of competing interest

The authors declare that they have no known competing financial interests or personal relationships that could have appeared to influence the work reported in this paper.
